# Similarly Potent Inhibition of Adenylyl Cyclase by P-Site Inhibitors in Hearts from Wild Type and AC5 Knockout Mice

**DOI:** 10.1371/journal.pone.0068009

**Published:** 2013-07-01

**Authors:** Joerg H. Braeunig, Frank Schweda, Pyung-Lim Han, Roland Seifert

**Affiliations:** 1 Institute of Pharmacology, Hannover Medical School, Hannover, Germany; 2 Institute of Physiology, University of Regensburg, Regensburg, Germany; 3 Department of Brain and Cognitive Science, Graduate School, Ewha Woman University, Seoul, Korea; Universidade Federal do Rio de Janeiro, Brazil

## Abstract

Adenylyl cyclase type 5 (AC5) was described as major cardiac AC isoform. The knockout of AC5 (AC5KO) exerted cardioprotective effects in heart failure. Our study explored the impact of AC5KO on mouse heart AC activities and evaluated putative AC5-selective inhibitors. In cardiac membranes from AC5KO mice, basal AC activity was decreased, while AC stimulation was intact. The putative AC5-selective P-site inhibitors SQ22,536 [9-(tetra-hydro-2-furanyl)-9H-purin-6-amine], vidarabine (9-β-D-arabinosyladenine) and NKY80 [2-amino-7-(2-furanyl)-7,8-dihydro-5(6H)-quinazolinone] inhibited recombinant AC5 more potently than AC2 and AC1, but selectivity was only modest (∼4-40-fold). These compounds inhibited cardiac AC from WT and AC5KO mice with similar potencies. In conclusion, AC regulation in AC5KO hearts was unimpaired, questioning the supposed dominant role of AC5 in the heart. Moreover, the AC inhibitors SQ22,536, NKY80 and vidarabine lack adequate selectivity for AC5 and, therefore, do not present suitable tools to study AC5-specific functions.

## Introduction

Signaling via the β_1_-adrenoreceptor (β-AR)-stimulatory G-protein (G_s_)- adenylyl cyclase (AC) cascade is the major mechanism to acutely improve cardiac performance [1]. Upon stimulation by β_1_-ARs, ACs synthesize the second messenger cAMP, which activates protein kinase A and subsequently leads to the phosphorylation of proteins regulating cardiac excitation-contraction coupling [1]. In acute heart failure (HF), signaling via β_1_-ARs is increased and initially preserves cardiac function, but its long-term activation in chronic HF promotes disease progression [2]. Accordingly, the treatment of chronic HF with β_1_-AR antagonists reduces morbidity and mortality, while positive inotropic drugs that increase β-AR signaling or cAMP levels such as catecholamines or phosphodiesterase inhibitors are detrimental [2], [3]. Therefore, inhibition of AC in the heart has been proposed as an alternative approach to β_1_-AR blockade [2]–[6].

Membranous ACs consist of nine isoforms (AC1-9) with AC5 being reported to be a major AC isoform in the heart [7]–[10]. Disruption of AC5 decreased basal and stimulated AC activities by ∼30–50% [11], [12]. Strikingly, AC5KO mice exhibited an extended lifespan and beneficial effects in models of HF [13]–[15]. Hence, AC5 inhibition could constitute an approach for the treatment of heart failure [2]–[6].

There are two major classes of AC inhibitors; compounds that act non-competitively, mimicking the cAMP · PP_i_ transition state [16], [17] (so-called P-site inhibitors) and compounds that compete with ATP at the catalytic site such as MANT [2′(3′)-*O*-(N-methylanthraniloyl]- substituted nucleotides [18]–[22]. The P-site inhibitors SQ22,536 [9-(tetra-hydro-2-furanyl)-9H-purin-6-amine, THFA], NKY80 [2-amino-7-(2-furanyl)-7,8-dihydro-5(6H)-quinazolinone] and vidarabine (9-β-D-arabinosyladenine, AraAde) exhibit selectivity for AC5 relative to ACs 1, 2 and 3 [6], [19], [23], [24]. Unfortunately, these putative AC5-selective inhibitors have not yet been comprehensively characterized with respect to their AC isoform-specificity and their usefulness as pharmacological tools for the study of AC5-specific cell functions [19]. For example, renin secretion is supposedly mediated by AC5 because it is inhibited by NKY80 [25], but knockout studies clarified that both AC5 and AC6 are involved [26]. The aim of the present study was to investigate the impact of AC5KO on mouse heart AC activities and to evaluate the selectivity of SQ22,536, NKY80 and vidarabine.

## Materials and Methods

### Materials

ATP, GTP, cAMP, creatine phosphokinase type 1 from rabbit muscle, phosphocreatine, IBMX (3-isobutyl-1-methylxanthine), MgCl_2_ (highest quality), (-)-isoproterenol, vidarabine, triethanolamine, dimethyl sulfoxide (DMSO), ethylenediaminetetraacetic acid (EDTA) and ethylene glycol tetraacetic acid (EGTA) were purchased from Sigma-Aldrich (St. Louis, MO, USA). SQ22,536, NKY80 and ammonium acetate were obtained from Merck (Darmstadt, Germany). Forskolin (FS) was supplied by LC Laboratories (Woburn, MA, USA). GTPγS (guanosine 5′-[γ-thio]triphosphate) was purchased from Biolog (Bremen, Germany). MANT-ITPγS (2′(3′)-*O*-(*N*-methylanthraniloyl)-inosine 5′-[γ-thio]triphosphate) was obtained from Jena Bioscience (Jena, Germany). Neutral alumina (N Super 1) was purchased from MP Biomedicals (Eschwege, Germany). [α-^32^P]ATP (3000 Ci/mmol) was from Hartmann Analytic (Braunschweig, Germany). Diethylpyrocarbonate (DEPC)-treated water was supplied by Applied Biosystems (Darmstadt, Germany). Rotiszint eco plus liquid scintillator was from Roth, (Karlsruhe, Germany). Baculoviruses encoding bovine AC1, rat AC2, and canine AC5 were kindly provided by Drs. A. G. Gilman (University of Texas Southwestern Medical Center, Dallas, TX, USA) and R. K. Sunahara (University of Michigan Medical School, Ann Arbor, MI, USA). *Sf9*-cells were from the American Type Cell Culture Collection (Rockville, MD, USA).

### AC5 Knockout Mice

All animal work was performed according to the guidelines for the Care and Use of Laboratory animals published by the US National Institutes of Health. Hearts were removed from mice that had been sacrificed by cervical dislocation under anesthesia (sevoflurane) and all efforts were made to minimize suffering. The protocol was approved by the Committee on the Ethics of Animal Experiments of the local government (Regierung der Oberpfalz, Permit Number: 54-2532.1-10/10). Homozygote wild type (WT) and AC5 knockout (AC5KO) mice were offspring of heterozygous breeder pairs originally generated by Lee et al. [27] and were backcrossed to C57BL/6J over more than 10 generations. The knockout of AC5 was confirmed by genotyping as described previously [27] and by the lack of cardiac AC5 mRNA expression in quantitative reverse transcription (qRT-PCR) experiments (specified under *Methods* for qRT-PCR below). Mice were housed in a temperature- and light-controlled environment according to the German animal protection law. Studies were performed with hearts from 16–20 week old male mice, which were shock frozen in liquid nitrogen after removal and subsequently stored at −80°C.

### Membrane Preparation of Mouse Hearts and *Sf9*-cells Expressing AC1, 2 and 5

Mouse heart membranes were prepared by differential centrifugation as previously described [28], [29], with minor modifications. All membrane preparation steps were performed at 4°C. Hearts were thawed in ice-cold homogenization buffer containing 5 mM Tris-HCl and 5 mM EDTA, pH 7.4. Homogenization was performed in a buffer volume amounting to 20-fold the heart tissue weight (w/v) with an automatic disperser (TK 18 basic ultra turrax, IKA, Staufen, Germany) applying 3 series of 10 sec each at 20,000 rpm with a 30 sec cooling period between each series. Organ debris was removed by an 8-min centrifugation at 500 *g*. The supernatant was sedimented by a 30-min centrifugation at 40,000 *g* and the resulting membrane pellet was resuspended in assay buffer consisting of 50 mM triethanolamine/HCl 1 mM EGTA and 5 mM MgCl_2_, pH 7.4. Membranes were resuspended with syringes in the sequence of 21 gauge, 27 gauge, shock-frozen in liquid nitrogen and stored at −80°C. *Sf9*-cell membranes expressing recombinant ACs 1, 2 and 5 were prepared according to established protocols for *Sf9-*infection [30] and -membrane preparation [31]. *Sf9*-cell membranes were finally resuspended and stored as described for mouse heart membrane preparation above using the same assay buffer. Membrane protein concentration was measured using the Bradford method [32] with the Roti-Quant quantitation assay (Roth, Karlsruhe, Germany).

### AC Activity Assay

AC activity assays were performed by a radiochemical method essentially described previously [29]. AC reactions were carried out in a total volume of 50 µl containing 20 µl of reaction buffer consisting of (final) 7 mM Mg^2+^, 40 µM ATP, 100 µM cAMP, 0.4 mg/ml creatine phosphokinase, 9 mM phosphocreatine, 100 µM IBMX, 0.5 to 1.0 µCi of [α-^32^P]ATP and 10 µl of one of the following variables: GTP, GTPγS, isoproterenol (ISO), forskolin (FS) or an AC inihibitor. AC inhibitors comprised SQ22,536, vidarabine, NKY80 and MANT-ITPγS. When assessing β-AR mediated AC activation, 10 µM GTP was added to the reaction buffer, while AC inhibition experiments were performed under stimulation with 100 µM FS and 10 µM GTPγS. Due to high hydrophobicity of FS and the AC inhibitors SQ22,536, vidarabine or NKY80, these substances were dissolved in DMSO. Final assay concentrations for DMSO in controls and experimental samples were 5% (v/v) for FS dose response-curves and 8% (v/v) for AC inhibition experiments. Assay tubes were pre-incubated for 2 min at 30°C and reactions were initiated by the addition of 20 µl of membranes (10 to 20 µg of protein/tube). Tubes were incubated for 15 min at 30°C or for a shorter period of 2 min in AC inhibition experiments to avoid nucleotide degradation (observed with MANT-nucleotides). The reaction progress was linear for observed time periods. Reactions were terminated by the addition of 20 µl of 2.2 N HCl, and denatured protein was sedimented by a 1-min centrifugation at 12,000 *g*. [^32^P]cAMP was separated from [α-^32^P]ATP by column chromatography with 1.4 g of neutral alumina as stationary phase. [^32^P]cAMP was eluted in 20-ml scintillation vials by adding 4 ml of 0.1 M ammonium acetate, pH 7.0. Blank values were <0.1% and substrate turnover was <2% of [α-^32^P]ATP added. Scintillation vials were filled up with 10 ml of double distilled water or Rotiszint eco plus liquid scintillator (Roth, Karlsruhe, Germany), mixed and Čerenkov radiation was determined.

### RNA Extraction and cDNA Synthesis

Total RNA from mice heart left ventricles (LVs) was extracted with the Nucleospin RNAII extraction kit (Macherey-Nagel, Düren, Germany) according to manufacturer’s guidelines. Homogenization was performed in lysis buffer amounting 30-fold of tissue weight (w/v) using the FastPrep24 homogenizer with Lysis Matrix D beads (MP Biomedicals, Eschwege, Germany) applying 4 series of 20-sec at speed setting 6.5 with a 5-min cooling period between each series. Contamination with genomic DNA was eliminated by a DNAse treatment step in the kit. RNA was eluted in a volume of 40 µl RNA-free diethylpyrocarbonate (DEPC)-treated water (Applied Biosystems, Darmstadt, Germany) and immediately used for cDNA synthesis. RNA integrity was checked by microcapillary electrophoretic RNA separation using Agilent 2100 bioanalyzer (Agilent, Santa Clara, CA, USA) providing even RIN values for RNA from WT (7.76±0.22 SD) and AC5KO (7.74±0.29 SD) mice hearts. First-strand cDNA was synthesized in a total volume of 80 µl containing 2 µg RNA, 2 µg Oligo(dT)18 Primer, 800 pg random hexamer primer, 16 µl reaction buffer of Revert Aid Transcriptase (5X), 80 U RiboLock RNase Inhibitor, 8 µl of dNTP mix (0.5 mM final each), 400 U Revert Aid reverse transcriptase (Fermentas, St. Leon-Rot, Germany) and DEPC-treated water. Reactions were incubated for 10 min at 37°C, followed by 60 min at 42°C and terminated with 10 min at 70°C on a primus 96 advanced thermal cycler (PEQLAB, Erlangen, Germany). cDNA was subsequently stored at −20°C.

### Quantitative Reverse Transcription PCR (qRT-PCR) Experiments

qRT-PCR experiments were performed employing predesigned TaqMan primer probe sets (Applied Biosystems, Darmstadt, Germany) listed in the Table S1. Details on qPCR experiments are documented in supplemental Figs. S1–S4 and Table S2. Reactions were conducted according to the manufacturer’s recommendations, which are common and standardized for all primer-probe sets. qRT-PCR reactions with a final volume of 20 µl contained 1X TaqMan Universal PCR Mastermix, 1X Gene Expression Assay (Applied Biosystems, Darmstadt, Germany) and 2 µl cDNA from mouse heart LVs as template. qRT-PCR runs were performed in 96-well optical plates as duplicates on a StepOnePlus real-time PCR system (Applied Biosystems, Darmstadt Germany). Thermal cycling conditions were 10 min at 95°C followed by 40 cycles of 15 s at 95°C and 1 min at 60°C. Analysis of PCR amplifications were executed with the qRT-PCR analysis software LinRegPCR [33]–[35] (Heart Failure Research Center, Amsterdam, Netherlands, V.12.12) from raw non-baseline corrected fluorescence data under the settings for cumulative fluorescence PCR-kinetics. The program uses linear regression on the log(fluorescence) per cycle in the exponential (log-linear) phase of the PCR reaction to calculate fluorescence thresholds, Ct-values (number of cycles to reach the fluorescence threshold), PCR efficiencies as well as an estimate of the initial template starting concentration expressed in arbitrary fluorescence units. The relative expression levels of components of the β-AR-G-protein-AC signaling cascade in LVs from WT vs. AC5KO were analyzed applying a Δ-Ct relative quantification model with PCR efficiency correction using the relative expression software tool REST [36] (V2.0.13) (M. Pfaffl., Technical University Munich, Qiagen, Hilden, Germany). Ct-values and amplicon-specific PCR efficiencies required for relative expression calculations were derived from prior analysis of PCR amplifications with LinRegPCR. Specificity of primer-probe sets was confirmed by agarose gel electrophoresis of respective PCR products, yielding specific bands of estimated sizes for each target (Fig. S1 and S2). In qRT-PCR reactions targeting AC5 no specific amplification was detected in cDNA samples from AC5KO mice (Fig. S1 and Fig. S3). Normalization was performed with hypoxanthine guanine phosphoribosyltransferase (HPRT) as a reference gene, which exhibited a stable expression in WT and AC5KO samples (Fig. S4).

### Miscellaneous

Linear and nonlinear regressions were performed with Prism V.5.01 (Graph Pad Software, San Diego, CA, USA). Statistical analysis was carried out using the students unpaired two tailed *t*-test and statistical comparison of best-fit parameters (bottom, logEC50 and top) of nonlinear regressions from concentration response curves were conducted with extra-sum-of-squares *F*-test (Prism). Statistical calculations of mRNA expression data were conducted with the relative quantification software tool REST (V2.0.13) based on a pairwise fixed reallocation randomization test with 2000 permutations and a cutoff for statistical significance of p<0.05. Experimental graphs were created with Prism except for figures presenting mRNA expression analysis, where the original graphical output from the REST software was modified to unify graphical appearance.

## Results and Discussion

### mRNA Expression Analysis of β-AR-G-protein-AC Signaling Components in AC5KO Hearts

**Figure 1:**
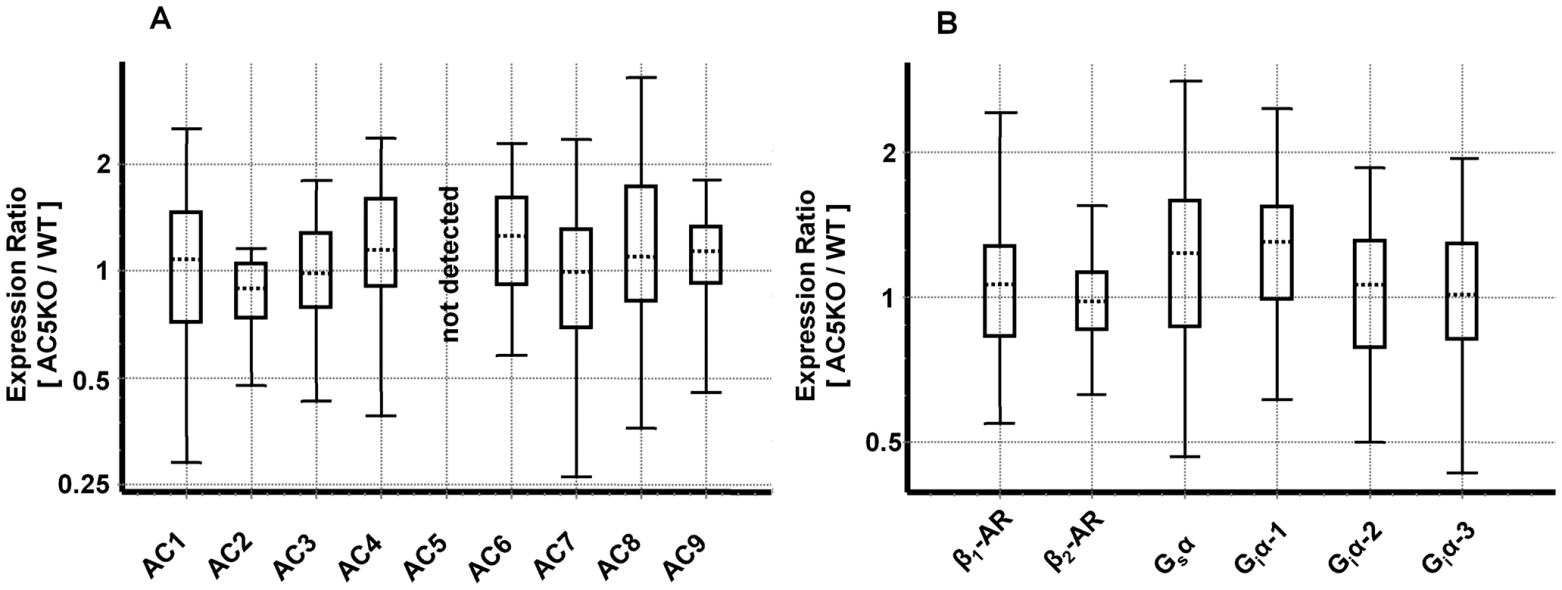
mRNA expression analysis of the β-AR-G-protein-AC signaling cascade in mouse hearts. Box and whisker plots show the gene expression ratios in left ventricles (LVs) from AC5KO- relative to that in WT mice obtained from qRT-PCR experiments described under *Methods*. **(A)** Relative mRNA expression of membranous ACs 1–9. AC5 mRNA was not detected in LVs from AC5KO hearts. **(B)** Relative mRNA expression of β-ARs 1 and 2, G_s_α and G_i_α 1–3. The lower and upper boundaries of the boxes represent the 25^th^ percentile and the 75^th^ percentile. The line within the boxes represents the median. The whiskers show the lower and upper 25% of observations. Data of each target **(A and B)** was obtained from qRT-PCR experiments with seven WT- and five AC5KO heart LVs performed in duplicates. There were no significant differences in the gene expressions of investigated targets between LVs from WT vs. AC5KO mice except for AC5.

We confirmed that AC5 was not expressed at the mRNA level in AC5KO hearts (Supplemental Figures S1 and S3). In contrast, all other membranous ACs were expressed in AC5KO hearts, and there were no differences between WT and AC5KO mice (Fig. 1A). Analysis of β-ARs and G-proteins did not reveal any altered expression in LV samples from AC5KO mice as well (Fig. 1B). qRT-PCR amplifications for AC2 and AC8 exhibited high Ct-values suggesting that mRNA expression of these isoforms was low. This finding is in accordance with previous reports [11], [29]. Relative expression ratios and characteristics of qRT-PCR experiments are presented in the Table S2. Due to poor isoform specificity of commercially available AC antibodies, the analysis of AC isoform expression at the protein level is very problematic [19], [29], [37], [38].

### AC Regulation in AC5KO Hearts

**Figure 2:**
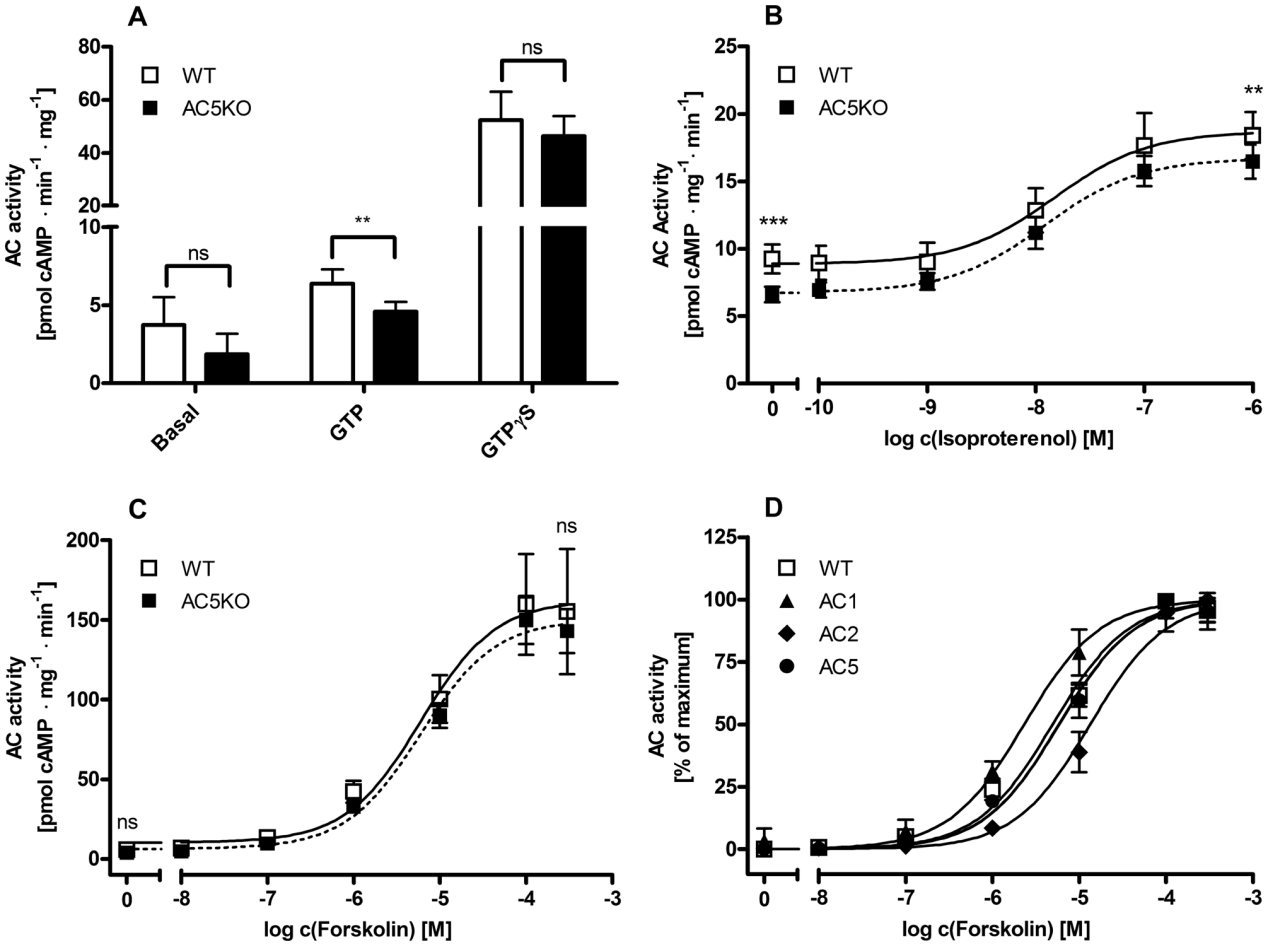
Stimulations of mouse heart AC and recombinant AC1, 2 and 5. AC activities were determined in membrane preparations of mouse hearts from wild type (WT) and AC5 knockout (AC5KO) mice or in membrane preparations of *Sf9*-cells expressing recombinant AC1, 2 and 5 respectively. **(A–C)** Mouse heart AC activities in WT vs. AC5KO mice. **(A)** Basal and G_s_α-stimulated AC activities. G_s_α was stimulated GTP (10 µM) or GTPγS (10 µM). **(B)** AC activities under β-AR-mediated stimulation with isoproterenol. Baseline AC activity represents AC activity under stimulation with GTP (10 µM). **(C)** Direct activation of mouse heart with forskolin (FS). **(D)** Comparison of FS mediated AC activation of mouse heart AC (WT) vs. recombinant AC1, 2 and 5. Each experiment assessing mouse heart AC activities was performed with five WT and AC5KO mice respectively. FS stimulated AC activities of recombinant AC isoforms were determined using three independent *Sf9*-cell membrane preparations. Data represents the means ± S.D. of experiments performed in triplicates **(A)** or duplicates in **(B–D)**. Not significant (ns) = p>0.05, **p<0.01, ***p<0.001.

Basal AC activity in hearts from AC5KO mice was reduced by ∼50% (Fig. 2A). With GTP, the difference between the groups amounted to 28%. There was no significant difference in AC stimulation by the direct G-protein activator GTPγS [39] (Fig. 2A). Stimulation of β-ARs with isoproterenol (ISO) increased AC activity in WT vs. AC5KO hearts with similar potencies and efficacies (Fig. 2B).

FS stimulated ACs in the order of potency: AC1> AC5 ∼ cardiac AC from WT>AC2 (Fig. 2D, Table 1). The respective EC_50-_values on AC1, 2, 5 and WT mouse heart AC were in good accordance to those determined previously [29]. There was no difference in EC_50_ between AC from WT and AC5KO (Fig. 2C).

**Table 1:** Potencies of FS on mouse heart AC and on AC1, 2 and 5.

	Mouseheart AC		RecombinantACs		
Parameter	WT	AC5KO	AC1	AC2	AC5
Potency of FS,EC_50_ [µM]	5.0	5.9 ns	2.3****	13.4****	5.8 ns
(95% C.I.)	(4.2–5.9)	(5.2–6.7)	(1.9–2.9)	(10.8–16.6)	(4.7–7.2)

EC_50_-values of FS on mouse heart AC from wild type (WT) or AC5 knockout (AC5KO) mice and on AC1, 2 and 5. EC_50_-values were calculated from nonlinear regression of respective FS concentration response curves **(** **Fig.1C and 1D** **)**. Data represents the means and the 95% confidence interval (C.I.) of experiments with five mouse hearts from WT and AC5KO mice respectively and of experiments with three independent membrane preparations of AC1, 2 and 5, performed in duplicates. logEC_50_-values were compared against the logEC_50_ derived from mouse heart AC from WT mice. Not significant (ns) = p>0.05,

**** p<0.0001.

**Figure 3:**
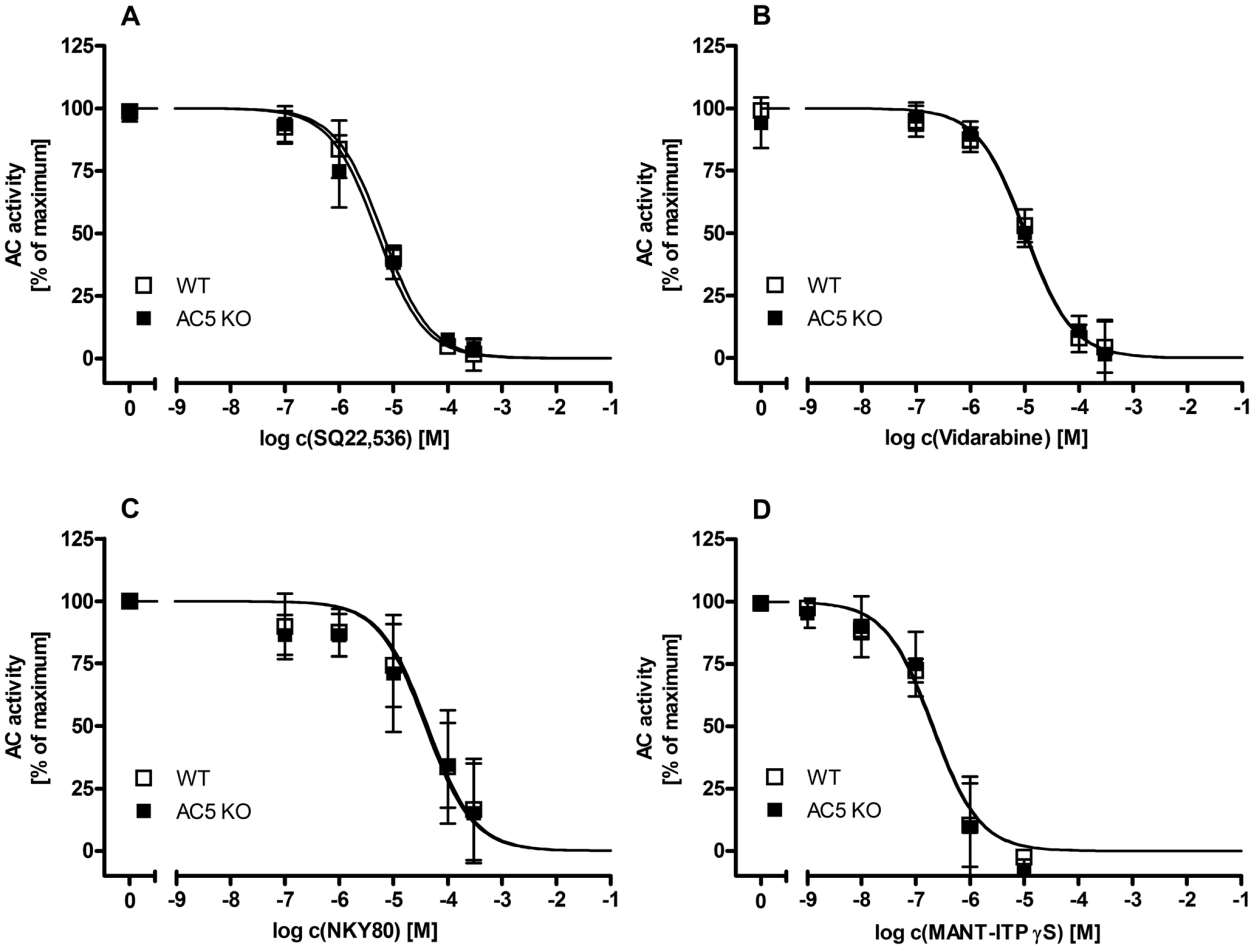
Inhibition of mouse heart AC with putative AC5-selective inhibitors. Concentration response curves of SQ22,536 **(A)**, vidarabine **(B)** and NKY80 **(C)** on mouse heart AC from wild type (WT) vs. AC5 knockout mice (AC5KO). MANT-ITPγS served as a potent reference AC inhibitor **(D).** Maximal AC activities represent AC activity under stimulation with FS (100 µM) and GTPγS (10 µM) in the absence of inhibitor. Absolute activites in WT vs. AC5KO hearts (pmol cAMP · mg^−1^ · min^−1^, ± SD): 136.7±46.51 vs. 120.1±31.4; p = 0.64. Data are the means ± S.D. of experiments with cardiac membrane preparations from three WT or AC5KO mice respectively performed in duplicates.

**Figure 4:**
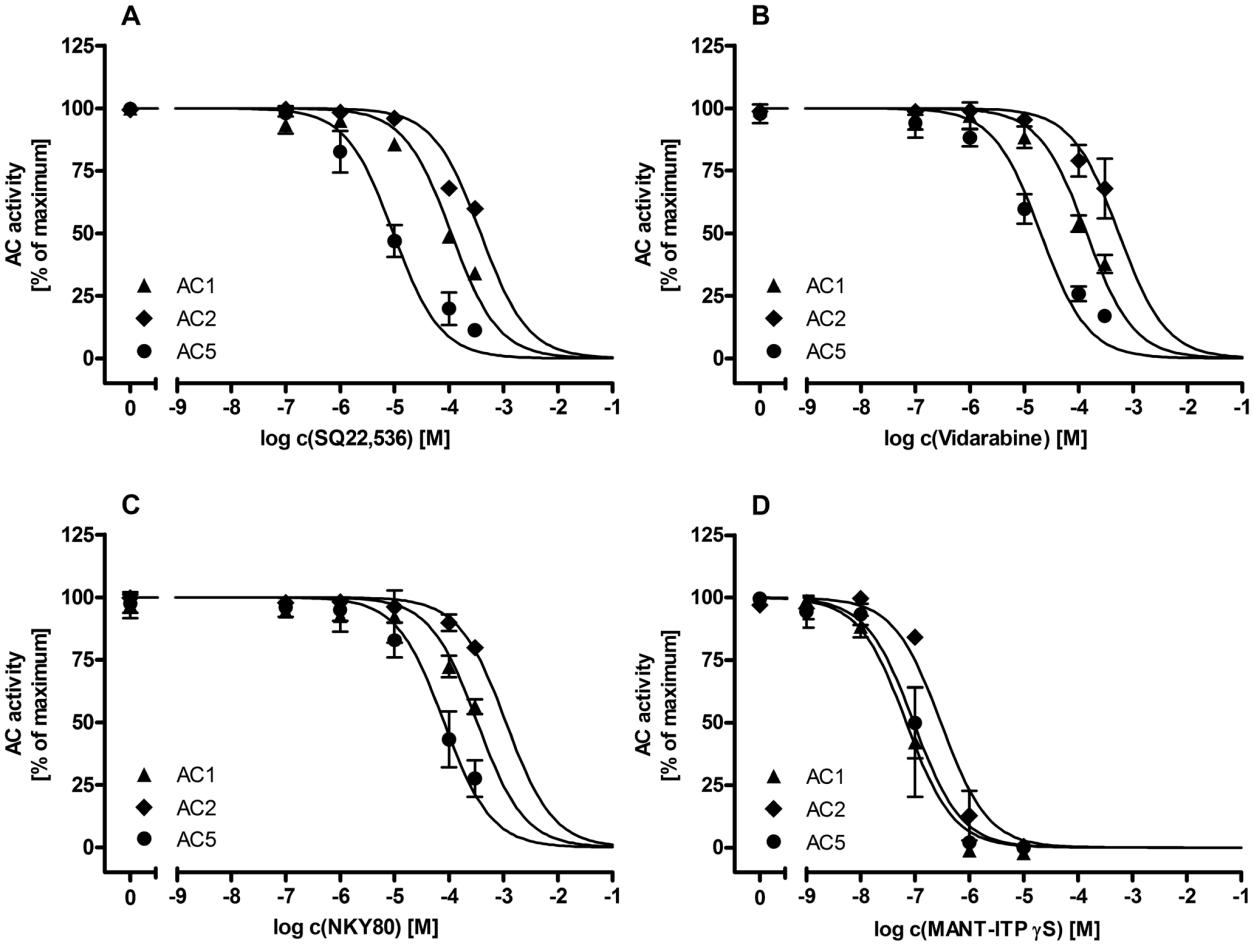
Inhibition of recombinant ACs with putative AC5-selective inhibitors. Comparison of concentration response curves of putative of AC5-selective inhibitors on recombinant AC1, 2 and 5. **(A)** SQ22,536, **(B)** vidarabine, **(C)** NKY80. MANT-ITPγS served as a potent reference AC inhibitor **(D).** Maximal AC activities represent AC activity under stimulation with forskolin (100 µM) and GTPγS (10 µM). Data are the means ± S.D. of experiments with three independent membrane preparations of *Sf9*-cells expressing AC1, 2 or 5 performed in duplicates.

None of the putative AC5-selective inhibitors inhibited cardiac AC from WT and AC5KO differentially (Fig. 3A–C). SQ22,536, NKY80 and vidarabine inhibited recombinant ACs in the order of potency: AC5> AC1> AC2 (Fig. 4A–C). Summaries of AC inhibitor potencies on cardiac AC from WT and AC5KO mice and on recombinant AC1, 2 and 5 are presented in Table 2. SQ22,536 exhibited the highest preference ratios for AC5 with 11.6-fold over AC1 and 40.2 -fold over AC2 (NKY80∶4.1 -fold over AC1 and 13.7 -fold over AC2; vidarabine: 6.9 -fold over AC1 and 27.3 -fold over AC2). MANT-ITPγS inhibited AC1 and AC5 with similar potency and ∼ 3-fold more potently than AC2 (Fig. 4D, Table 2). There was no difference in EC_50_ for MANT-ITPγS between AC from WT and AC5KO (Fig. 3D).

**Table 2:** Inhibitor potencies on mouse heart AC and on AC1, 2 and 5.

AC Inhibitor	Mouse heart AC		Recombinant ACs		
IC_50_ [µM]; (C.I.95%)	WT	AC5KO	AC1	AC2	AC5
SQ22,536	6.3	5.0 ns	110.1****	381.0 ns	9.5**
	(4.8–8.3)	(3.5–7.2)	(84.6–143.4)	(299.3–484.9)	(6.1–14.7)
NKY80	42.5	38.5 ns	330.1****	1112.0****	81.1 ns
	(22.4–80.6)	(19.7–75.4)	(249.1–437.5)	(931.5–1326.0)	(61.2–107.5)
Vidarabine	10.5	10.0 ns	138.2****	544.6****	20.0 ns
	(8.0–13.6)	(7.2–13.9)	(113.1–168.9)	(420.5–705.3)	(13.7–29.1)
MANT-ITPγS	0.2	0.2 ns	0.07****	0.30****	0.09*
	(0.14–0.29)	(0.12–0.36)	(0.05–0.10)	(0.21–0.42)	(0.07–0.13)

IC_50_-values of putative AC5-selective inhibitors SQ22,536, NKY80 and vidarabine and of the reference inhibitor MANT-ITPγS on mouse heart AC from wild type (WT) or AC5 knockout mice (AC5KO) and on recombinant AC1, 2 and 5. IC_50_-values were calculated from concentration response curves demonstrated in Fig. 3 and 4 and are the means and the 95% confidence intervals (C.I.) of three independent experiments performed in duplicates. logIC_50_-values were compared against the logIC_50_ derived from mouse heart AC from AC5KOmice. Not significant (ns) = p>0.05,

* p<0.05,

** p<0.01,

*** p<0.001,

**** p<0.0001.

### Conclusions

Under stimulated conditions AC5 contributed little to total AC activity in crude heart membranes. Previous studies reported a larger contribution of AC5 to total AC activity in the heart [11], [12]. Mice investigated by Tang et al. and us were derived from the same AC5KO-model back-crossed to the C57BL/6 strain [12], [27]. However, mice studied by Tang et al. [12] were younger than those currently investigated by us (2.5 month vs. ∼3.5–4.5 month). We cannot exclude that differences in age caused differences in AC5 expression [37], [40], [41]. Crude cardiac membrane preparations contain cardiomoycytes, fibroblasts, endothelial cells and vascular smooth muscle cells. However, similar changes in mouse heart AC activity due to disruption of AC5 were reported for crude cardiac membrane- and pure cardiomyocyte preparations [12], [42].

Quantitative data about mRNA expression levels of AC5 and AC6 in human hearts are conflicting [43], [44]. In adult murine cardiomyocytes, qRT-PCR readily detected mRNA expression for AC4, 5, 6 and 9, a weak expression for AC7 and no expression for AC1, 2, 3 and 8 [45]. Our qRT-PCR experiments with LV tissue suggest that the AC isoform expression pattern was comparable to that in isolated cardiomyocytes.

SQ22,536, NKY80 and vidarabine exhibited a moderate preference for AC5 over AC1 and AC2 and inhibited AC in WT and AC5KO heart with similar potency. Even the preference ratios of the most ‘selective’ AC5 inhibitor SQ22,536 over AC1 and AC2 were not compelling considering that AC1, AC2 and AC5 belong to different AC families [3], [7], [8] and are likely to possess more different pharmacological properties than members of the same AC family [19]. Vidarabine was employed to mimic cardioprotective effects induced by AC5 disruption in a model of chronic catecholamine stress [42]. The selectivity of vidarabine for AC5 was supported by inhibition experiments on mouse heart AC from knockout models [42]. At the moment we cannot provide a convincing explanation for the striking discrepancies between our results and recently published data [42]. It will be essential for the development of truly AC isoform-selective inhibitors that compounds are examined on all ACs (AC1-9), which has not been achieved so far [19].

The low potency and poor solubility of P-site inhibitors is a serious concern [3], [5], [19]. Increasing the concentration of organic solvents such as DMSO, which is required to ensure solubilization at higher P-site inhibitor concentrations, limits application of these compounds. Nucleoside-based P-site inhibitors such as SQ22,536 and vidarabine, which is also a virustatic drug [46], are very likely to influence DNA polymerases and purine metabolism at concentrations that inhibit AC [3], [5], [19], [23]. SQ22,536 inhibits extracellular signal-regulated kinase signal transduction in an AC-independent manner [47]. Off-target effects of NKY80 have not yet been examined.

Collectively, AC inhibitors SQ22,536, NKY80 and vidarabine demonstrated only moderate selectivity for AC5 and do not constitute appropriate tools to study AC5-specific functions. In our hands, AC5 was not the major cardiac AC isoform in mice. The concept of AC5 inhibition for the treatment of heart failure requires further evaluation.

## Supporting Information

Figure S1 **Gel electrophoresis of PCR products for AC1-9.** PCR products were amplified from mouse heart cDNA described under *Methods* using TaqMan primer-probe sets listed in Table S1. Primer-probe sets for AC1-9 produced specific bands of appropriate sizes, which are indicated above. In AC5KO mice the specific band for the AC5 amplicon at 85 base pairs (bp) was not detected. The DNA ladder (GeneRuler 50 bp) was applied on the left (0.5 µg) and right (1 µg) side of the gel. In order to obtain bands of roughly similar intensity the volume of loaded PCR reaction sample of amplifications for AC1-9 was adjusted differently (10 µl for AC1 and AC2; 5 µl for AC3, and AC8, 3 µl for AC4, AC5 for WT and AC5KO, AC6 a and b, AC7, AC9).
(TIF)

Figure S2 **Gel electrophoresis of PCR products for G-proteins and β-adrenoceptors (β-ARs).** PCR products were amplified from mouse heart cDNA obtained from qRT-PCR experiments using TaqMan primer-probe sets listed in Table S1. Primer-probe sets produced specific bands of appropriate sizes, which are indicated above. 5 µl of PCR product and 0.5 µg (left) or 1 µg (right) of 50 bp DNA ladder were loaded per lane.
(TIF)

Figure S3 **Amplification plots of qRT-PCR experiments targeting AC5.** Screenshot of the LinRegPCR program analysis window, which shows amplification curves of qRT-PCR experiments targeting AC5 in cDNA samples of left ventricles (LVs) from wild type (n = 7) and AC5 knockout (AC5KO, n = 5) mice performed in duplicates. In cDNA samples of AC5KO mice LVs no specific amplification for AC5 was detected.
(TIFF)

Figure S4 **mRNA expression stability of the housekeeping gene hypoxanthine guanine phosphoribosyl transferase (HPRT) in left ventricles of wild type (WT) vs. AC5 knockout (AC5KO) hearts.** Box and whisker plots show the Ct-values of HPRT obtained in qRT-PCR experiments using total RNA from seven WT and five AC5KO animals measured in duplicates. The upper and lower boundaries represent the 25^th^ and 75^th^ percentile. The line within the boxes represents the median. The whiskers show the maximum and minimum observation. Mean Ct-values ± SD for WT vs. AC5KO were: 24.81±0.2642 vs. 24.79±0.1858. Comparing the mean Ct-values with the Student’s unpaired two tailed *t*-test produced a p-value of 0.8890, which was considered not significant (*p*>0.05).
(TIF)

Table S1 (DOC)

Table S2 (DOC)
