# Supplementary figures and images for: Similarly Potent Inhibition of Adenylyl Cyclase by P-Site Inhibitors in Hearts from Wild Type and AC5 Knockout Mice
Source: PLoS One. 2013 Jul 1;8(7):e68009. doi: 10.1371/journal.pone.0068009 (PMC3698094; doi:10.1371/journal.pone.0068009)

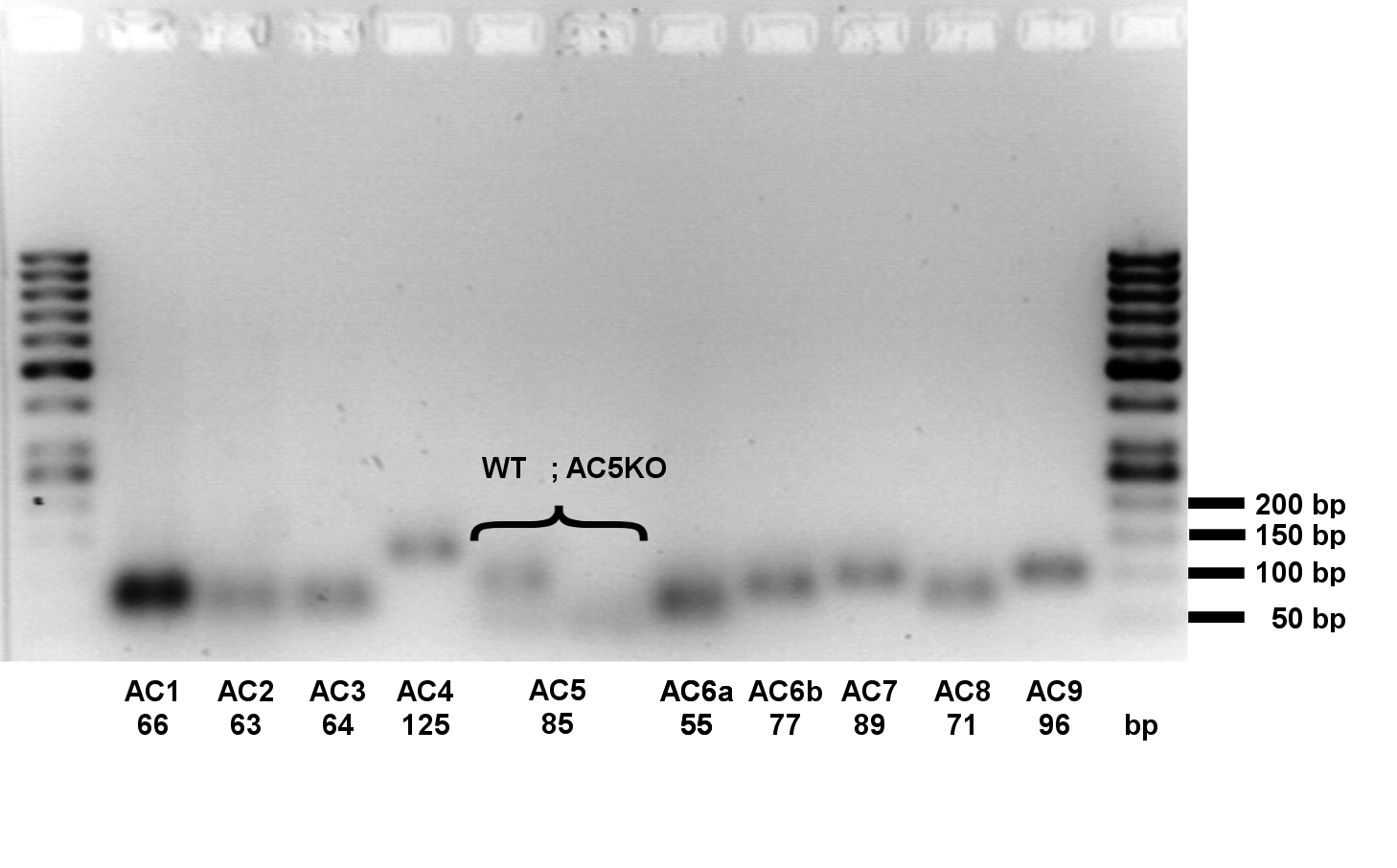

Supplement: Figure S1 — Gel electrophoresis of PCR products for AC1-9. PCR products were amplified from mouse heart cDNA described under Methods using TaqMan primer-probe sets listed in Table S1. Primer-probe sets for AC1-9 produced specific bands of appropriate sizes, which are indicated above. In AC5KO mice the specific band for the AC5 amplicon at 85 base pairs (bp) was not detected. The DNA ladder (GeneRuler 50 bp) was applied on the left (0.5 µg) and right (1 µg) side of the gel. In order to obtain bands of roughly similar intensity the volume of loaded PCR reaction sample of amplifications for AC1-9 was adjusted differently (10 µl for AC1 and AC2; 5 µl for AC3, and AC8, 3 µl for AC4, AC5 for WT and AC5KO, AC6 a and b, AC7, AC9). (TIF) [file pone.0068009.s001.tif]

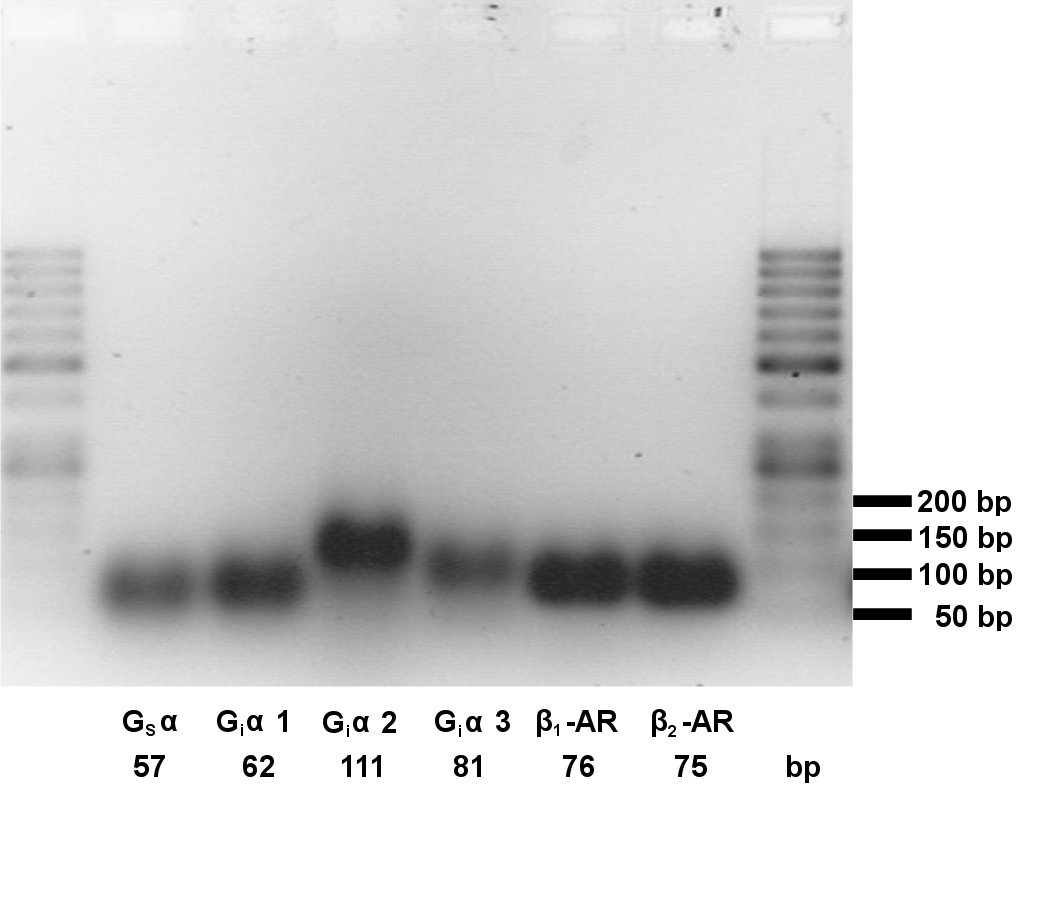

Supplement: Figure S2 — Gel electrophoresis of PCR products for G-proteins and β-adrenoceptors (β-ARs). PCR products were amplified from mouse heart cDNA obtained from qRT-PCR experiments using TaqMan primer-probe sets listed in Table S1. Primer-probe sets produced specific bands of appropriate sizes, which are indicated above. 5 µl of PCR product and 0.5 µg (left) or 1 µg (right) of 50 bp DNA ladder were loaded per lane. (TIF) [file pone.0068009.s002.tif]
