# Supplementary material for: Similarly Potent Inhibition of Adenylyl Cyclase by P-Site Inhibitors in Hearts from Wild Type and AC5 Knockout Mice
Source: PLoS One. 2013 Jul 1;8(7):e68009. doi: 10.1371/journal.pone.0068009 (PMC3698094; doi:10.1371/journal.pone.0068009)
Supplement: Table S1 — (DOC) [file pone.0068009.s005.doc]

| **Supplemental Table S1. Primer-probe sets used for quantitative reverse transcription PCR (qRT-PCR) experiments**. Primer-probe sets listed below were supplied by Applied Biosystems (TaqMan expression assays, Applied Biosystems, Darmstad, Germany). | | | | | | | |
| --- | --- | --- | --- | --- | --- | --- | --- |
| **Gene Name** | **Gene Symbol** | **Accession#** | **Reference Sequence** | **Translated Protein** | **Exon Boundary** | **Assay Location** | **Amplicon Length** |
| Adenylyl cyclase 1 | Adcy1 | Mm01187829_m1 | NM_009622.1 | NP_033752.1 | 19-20 | 3166 | 66 |
| Adenylyl cyclase 2 | Adcy2 | Mm00467874_m1 | NM_1535342 | NP_705762.2 | 4-5 | 978 | 63 |
| Adenylyl cyclase 3 | Adcy3 | Mm00460371_m1 | NM_001159536.1 | NP_001153008.1 | 11-12 | 2462 | 64 |
| Adenylyl cyclase 4 | Adcy4 | Mm00475491_m1 | NM_080435.1 | NP_536683.1 | 11-12 | 1639 | 125 |
| Adenylyl cyclase 5 | Adcy5 | Mm01343347_m1 | NM_001012765.4 | NP_001012783.3 | 1-2 | 1601 | 85 |
| *Adenylyl cyclase 6 ; a) | Adcy6 | Mm00475772_m1 | NM_007405.2 | NP_031431.2 | 2-3 | 1013 | 55 |
| *Adenylyl cyclase 6 ; b) | Adcy6 | Mm00475785_m1 | NM_007405.2 | NP_031431.2 | 16-17 | 2777 | 77 |
| Adenylyl cyclase 7 | Adcy7 | Mm00545780_m1 | NM_001037723.3 | NP_001032812.2 | 16-17 | 2346 | 89 |
| Adenylyl cyclase 8 | Adcy8 | Mm00507722_m1 | NM_009623.2 | NP_033753.2 | 4-5 | 2538 | 71 |
| Adenylyl cyclase 9 | Adcy9 | Mm00507743_m1 | NM_009624.2 | NP_033754.2 | 9-10 | 2913 | 96 |
| β_1_-adrenergic receptor | Adrb1 | Mm00431701_s1 | NM_007419.2 | NP_031445.2 | 1-1 | 252 | 76 |
| β_2_-adrenergic receptor | Adrb2 | Mm02524224_s1 | NM_007420.2 | NP_031446.2 | 1-1 | 1955 | 75 |
| G-protein alpha stimulating | Gnas | Mm01242435_m1 | NM_001077507.1 | NP_001070975.1 | 4-5 | 2609 | 57 |
| G-protein alpha inhibiting 1 | Gnai1 | Mm01165301_m1 | NM_010305.1 | NP_034435.1 | 4-5 | 705 | 62 |
| G-protein alpha inhibiting 2 | Gnai2 | Mm01232052_g1 | NM_008138.4 | NP_032164.2 | 8-9 | 1221 | 111 |
| G-protein alpha inhibiting 3 | Gnai3 | Mm00802670_m1 | NM_010306.2 | NP_034436.1 | 3-4 | 413 | 81 |
| Hypoxanthine guanine phosphoribosyl transferase | Hprt | Mm00446968_m1 | NM_013556.2 | NP_038584.2 | 6-7 | 630 | 65 |
| * Two different primer-probe set for mRNA analysis targeting AC6 designated a) and b). qRT-PCR experiments with both primer-probe sets resulted in very similar mRNA expression ratios (mean difference AC5KO/WT < 10%). mRNA expression data shown in the main article (Fig. 1A) and in the Supplemental Table S2 was obtained with AC6 primer-probe set a). | | | | | | | |
