# Supplementary material for: Similarly Potent Inhibition of Adenylyl Cyclase by P-Site Inhibitors in Hearts from Wild Type and AC5 Knockout Mice
Source: PLoS One. 2013 Jul 1;8(7):e68009. doi: 10.1371/journal.pone.0068009 (PMC3698094; doi:10.1371/journal.pone.0068009)
Supplement: Table S2 — (DOC) [file pone.0068009.s006.doc]

| **Supplemental Table S2. mRNA expression ratios of β-AR-G-protein-AC signaling components in wild type (WT) vs. AC5 knockout (AC5KO) mouse heart left ventricles measured by quantitative reverse transcription (qRT-PCR).** Expression ratios, respective 95% confidence intervals (C.I. 95%) and p-values were calculated using the relative expression software tool REST [1] (M. Pfaffl., Technical University Munich, Qiagen, Hilden, Germany, V2.0.13) based on cycles thresholds (Ct-values) and PCR efficiencies determined with the quantitative reverse transcription PCR analysis software LinRegPCR [2,3] (Heart Failure Research Center, Amsterdam, Netherlands, V.12.12). LinRegPCR analysis was performed from raw (non-baseline corrected) fluorescence data under the settings for cumulative fluorescence PCR-kinetics. Calculations were performed with mean PCR efficiencies per amplicon, comprising all samples in which the same pair of primers was used. Normalization was done with hypoxanthine guanine phosphoribosyltransferase (HPRT) as a reference gene. Data was obtained from experiments with seven WT and five AC5KO hearts performed in duplicates. Statistical calculations were conducted with the software REST based on a pairwise fixed reallocation randomization test with 2000 permutations and a cutoff for statistical significance of p<0.05. Notably, the amplification curve analysis method conducted with LinRegPCR generally determines lower PCR efficiencies as compared to standard curve based methods [4]. | | | | | | |
| --- | --- | --- | --- | --- | --- | --- |
| **Target Gene** | **Relative Expression** | | | **PCR Efficiency (+/- SD)** | **Mean Ct-values (+/- SD)** | |
|  | **Ratio (AC5KO/WT)** | **95% C.I.** | ***p*** |  | **WT** | **AC5KO** |
| HPRT | 1.000 | - | - | 1.727 (0.025) | 24.807 (0.264) | 24.788 (0.186) |
| AC1 | 1.012 | 0.367 - 2.241 | 0.956 | 1.775 (0.030) | 30.818 (0.880) | 30.779 (0.651) |
| AC2 | 0.861 | 0.513 - 1.155 | 0.174 | 1.743 (0.043) | 35.063 (0.420) | 35.313 (0.416) |
| AC3 | 0.954 | 0.434 - 1.739 | 0.771 | 1.795 (0.019) | 30.565 (0.582) | 30.628 (0.531) |
| AC4 | 1.128 | 0.439 - 2.311 | 0.560 | 1.740 (0.021) | 28.512 (0.678) | 28.276 (0.715) |
| *AC5 | - | - | - | 1.927 (0.049) | 26.759 (0.448) | - |
| AC6 | 1.219 | 0.583 - 2.086 | 0.239 | 1.757 (0.021) | 24.546 (0.434) | 24.175 (0.580) |
| AC7 | 0.910 | 0.327 - 2.011 | 0.695 | 1.803 (0.015) | 29.334 (0.670) | 29.477 (0.750) |
| AC8 | 1.138 | 0.410 - 3.490 | 0.621 | 1.801 (0.010) | 33.192 (0.886) | 32.955 (0.621) |
| AC9 | 1.052 | 0.533 - 1.753 | 0.733 | 1.841 (0.037) | 27.630 (0.460) | 27.529 (0.502) |
| β_1_-AR | 1.044 | 0.558 - 2.426 | 0.784 | 1.851 (0.024) | 27.283 (0.529) | 27.196 (0.421) |
| β_2_-AR | 0.978 | 0.658 - 1.453 | 0.816 | 1.759 (0.024) | 28.195 (0.383) | 28.215 (0.250) |
| G_s_α | 1.185 | 0.496 - 2.471 | 0.405 | 1.856 (0.034) | 22.560 (0.693) | 22.268 (0.470) |
| G_i_α 1 | 1.264 | 0.671 - 2.286 | 0.173 | 1.892 (0.027) | 28.558 (0.514) | 28.175 (0.372) |
| G_i_α 2 | 1.013 | 0.503 - 1.767 | 0.942 | 1.734 (0.045) | 22.987 (0.419) | 22.945 (0.590) |
| G_i_α 3 | 1.027 | 0.528 - 1.931 | 0.867 | 1.858 (0.035) | 26.089 (0.596) | 26.029 (0.404) |
| * There was no specific amplification for AC5 detected in samples from AC5KO hearts. Accordingly, mean PCR efficiencies for the AC5 amplicon represent WT heart samples only. | | | | | | |

1. Pfaffl MW, Horgan GW, Dempfle L (2002) Relative expression software tool (REST) for group-wise comparison and statistical analysis of relative expression results in real-time PCR. Nucleic acids research 30: e36.

2. Ruijter JM, Ramakers C, Hoogaars WMH, Karlen Y, Bakker O, et al. (2009) Amplification efficiency: linking baseline and bias in the analysis of quantitative PCR data. Nucleic acids research 37: e45. doi:10.1093/nar/gkp045.

3. Tuomi JM, Voorbraak F, Jones DL, Ruijter JM (2010) Bias in the Cq value observed with hydrolysis probe based quantitative PCR can be corrected with the estimated PCR efficiency value. Methods (San Diego, Calif) 50: 313–322. doi:10.1016/j.ymeth.2010.02.003.

4. Ruijter JM, Pfaffl MW, Zhao S, Spiess AN, Boggy G, et al. (2012) Evaluation of qPCR curve analysis methods for reliable biomarker discovery: Bias, resolution, precision, and implications. Methods (San Diego, Calif) 59: 32–46. doi:10.1016/j.ymeth.2012.08.011.
